# Therapeutic effects and mechanism analysis of Paeonia lactiflora extract (PLE) in menopausal rats with hot flashes

**DOI:** 10.3389/fphar.2025.1587885

**Published:** 2025-07-22

**Authors:** Weilin Cui, Tingting Song, Dongmei Gao, Xiaoyu Wang, Ya Sun, Liyu Fu, Yichao Han, Jieqiong Wang

**Affiliations:** ^1^ Faculty of Pharmacy, Shandong University of Traditional Chinese Medicine, Jinan, China; ^2^ The 970th Hospital of the Joint Service Support Force of the Chinese People’s Liberation Army, Weihai, China; ^3^ Teaching and Research Office of Basic Theory of Traditional Chinese Medicine, College of Traditional Chinese Medicine, Shandong University of Traditional Chinese Medicine, Jinan, China; ^4^ Department of Graduate, Shandong University of Traditional Chinese Medicine, Jinan, China; ^5^ Scientific Research Achievements Transformation Department, Office of Academic Research, Shandong University of Traditional Chinese Medicine, Jinan, China

**Keywords:** paeonia lactiflora extract, menopausal syndrome, transcriptomics, hypothalamus, hot flashes

## Abstract

**Ethnopharmacological relevance:**

Menopausal syndrome (MPS) is a symptom of physical and psychosomatic abnormalities that women may face around the time of menopause. Hot flashes are the main symptom. Paeonia lactiflora extract (PLE) is the active ingredient extracted from *Radix Paeonia alba*. It can be used to treat MPS, such as hot flashes. However, its pharmacologic mechanism is unclear.

**Aim of the study:**

This study aims to comprehensively evaluate the effect of PLE on menopausal hot flashes, and to analyze the mechanism of action of PLE in the treatment of menopausal hot flashes from the perspective of neural pathways, to provide a research strategy and experimental basis for the study of similar new drugs and the pathogenesis of MPS.

**Materials and methods:**

First, we screened menopausal rats through the natural aging model. After 14 days of therapeutic drug gavage, a menopausal hot flashes model was induced in menopausal rats by gavage with thyroid tablet suspension (160 mg/kg) for 14 days. The changes in facial and tail temperature of rats in each group were observed; the behavioral characteristics of rats in each group were followed by an open field test, elevated plus maze and aggressive behavior test; the contents of estradiol, luteinizing hormone, follicle stimulating hormone, 5-hydroxytryptamine, cyclic guanosine monophosphate and cyclic adenosine monophosphate were detected by ELISA; the pathological changes of the uterus were detected by HE staining method. Combined with transcriptomics technology, high-throughput transcriptome sequencing was performed on the hypothalamus of control, model and PLE (160 mg/kg) group, and differential gene analysis between control and model groups, and PLE (160 mg/kg) group and model group was performed using DESeq2. qRT-PCR and Western blot were used to further validate the candidate core genes.

**Results:**

PLE improved the mental status of model rats and reduced the abnormal tail temperature elevation in model rats. In addition, PLE had the effect of increasing the estradiol content and decreasing the luteinizing hormone content in the serum of rats, and the administration of 160 mg/kg of PLE also significantly increased the 5-hydroxytryptamine content in the serum of rats. In terms of pathological manifestations, the model rats had significantly thinner endometrial thickness, looser tissues and reduced integrity, while the rats intervened by PLE treatment had significantly thicker endometrium and more regularly arranged tissue structure. Therefore, it can be determined that PLE has a good pharmacological basis for the treatment of menopausal hot flashes. The transcriptomic analysis showed that 210 genes were significantly altered in the control and drug administration groups together, and the candidate core genes related to neuroendocrine were screened out based on the comprehensive literature and previous studies, and it was further found that PLE may achieve ASIC4, cplx1, mRNA expression levels, and Tac3, Tacr3 protein expression levels by up-regulating neuroprotective effects, thereby restoring the normal neuroendocrine environment of menopausal hot flashes in rats.

**Conclusion:**

PLE can effectively alleviate thyroid tablet-induced menopausal hot flashes, and the mechanism may be related to the regulation of abnormal expression of ASIC4, cplx1, GnRH1, Tac3, and Tacr3 in the hypothalamus, thereby restoring the imbalanced hypothalamic-pituitary-gonadal axis.

## Graphical Abstract

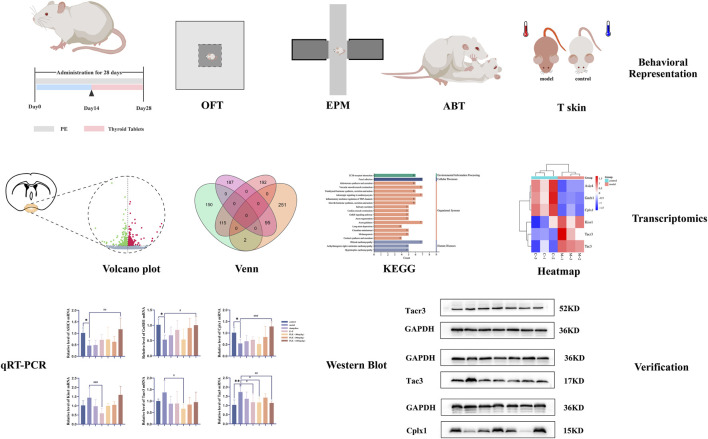

## 1 Introduction

Menopausal syndrome (MPS) refers to a group of syndromes in which women experience fluctuations or decreases in estrogen around the time of menopause due to the decline of ovarian function, which in turn leads to a series of disorders of the autonomic nervous system, accompanied by psychological symptoms, such as hot flashes and sweating, irritability, insomnia and forgetfulness, back pain, etc.(Li et al., 2019; Minkin, 2019; Santoro et al., 2015).

Hot flashes are a characteristic symptom of MPS (Gracia and Freeman, 2018).Studies have found that more than 80% of women may experience hot flashes during menopause, of varying duration, usually with a sudden, intense warmth of the skin on the face, neck and chest, accompanied by profuse sweating, and possibly fidgeting and irritability (Freedman, 2014). The frequency and severity of hot flashes affect women’s work, life and sleep, causing a lot of physical and psychological pain to women. In addition, studies have found that frequent hot flashes also increase the risk of cardiovascular disease in women in middle and old age (Thurston et al., 2021). Therefore, the research on this disorder has become one of the hot issues worldwide.

Wang W. et al. (2022) performed RNA-seq on the hypothalamus of OVX mice and found differences in the expression of genes involved in thermoregulation, feeding, sleep, homeostasis and endocrine regulation in the hypothalamus 8 weeks after ovariectomy, suggesting a possible role in the pathogenesis of menopausal syndrome. Bacon et al. (2019) applied transcriptomics to discover transcriptional and epigenomic changes during endocrine aging in rats entering menopause, with hypothalamic aging preceding the onset of menopause. This provides evidence for a critical period of neuroendocrine aging in the female brain prior to ovarian failure. This suggests that damage to the neural circuits within the hypothalamus is one of the key links in the pathogenesis of menopausal hot flashes, while the exact mechanism is unclear.

Currently, there are many treatments used to relieve hot flashes, but their efficacy varies. Hormone replacement therapy (HRT) is an effective treatment for hot flashes (Hill et al., 2016; Talaulikar, 2022). Patients are treated with estrogen and progesterone supplementation, which is useful for hot flashes and menopause-related symptoms, but its treatment greatly increases the risk of venous thromboembolism, breast cancer, and endometrial hyperplasia (Thurston et al., 2021). Therefore, it is important to find effective and safe natural drug alternative treatments.

Paeonia lactiflora extract (PLE) is extracted from *Radix Paeoniae Alba*, refined, concentrated and dried to produce a natural extract. Previous studies by our group have shown that PLE has a good effect on depression and anxiety induced by chronic restraint stress stimulation and electrical stimulation in rats (Song et al., 2016; Wang et al., 2020). The combination formula of extracts of ligustrum lucidum and paeonia lactiflora significantly increased the E2 content and decreased the GnRH, FSH and LH content in the serum of ovariectomized rats, thus improving the symptoms associated with menopausal syndrome (Cui et al., 2022). In addition, the combination formula of extracts of ligustrum lucidum and paeonia lactiflora was also found to increase estrogen receptor protein content in the uterus and ovaries of naturally aging mice (Song et al., 2022). However, the direct protective function and mechanism of action of PLE with menopausal hot flashes is unclear, and another important question is whether PLE has an effect on hypothalamic neural circuits.

Therefore, this study was conducted to evaluate the effect of PLE on the treatment of hot flashes in MPS from the perspective of the menopausal hot flashes model by observing the physiological indexes such as mental status, facial temperature and tail temperature changes of rats before and after administration; behavioral indexes such as open field test (OFT), elevated plus maze (EPM) and aggressive behavior test (ABT); microscopic indexes such as estradiol (E2) content, follicle stimulating hormone (FSH) content, luteinizing hormone (LH) content, 5-hydroxytryptamine (5-HT) content, cyclic adenosine monophosphate (cAMP), cyclic guanosine monophosphate (cGMP) content and pathological examination of uterus. In addition, the effect of PLE on hot flashes, the main symptom of menopausal syndrome, was evaluated by combining transcriptomic techniques and exploring the changes in the hypothalamus of rats in a model of menopausal hot flashes, and constructing a biochemical analysis-effective molecular network of PLE for the treatment of hot flashes. This study is expected to provide a research strategy and experimental basis for similar new drug research and research on the pathogenesis of menopausal hot flashes.

## 2 Materials and methods

### 2.1 Animals

This study used 90 SPF female, healthy, non-pregnant SD rats aged 9 months, 15 SPF female, healthy, non-pregnant SD rats aged 3 months, and 40 SPF female, healthy, non-pregnant Wistar rats aged 3 months. All animals were produced by Beijing Vital River Laboratory Animal Technology Co., Ltd. The animals were adapted for 1 week and kept under a constant temperature of 20–26°C, and relative humidity of 50%–60%, and 12/12 h light/dark cycle (20:00 lights on, 8:00 lights out). Except during the experiment, they are free to eat and drink. The Experimental Animal Ethics Committee approves the experimental scheme of the Shandong University of Traditional Chinese Medicine (SDUTCM20201030003).

### 2.2 Preparation of PLE

PLE used in this study were provided by Lunan Pharmaceutical Group Corporation. (Shandong, China), and the voucher specimen was also kept in the Shandong University of Traditional Chinese Medicine (Shandong, China). Batch No.21PF01. The main technological process: take the pieces of white peony root, add 8,6,4 times the amount of 30% ethanol, soak 30 min before extraction, extract three times, 2 h each time, and combine the extract. The extract was concentrated under reduced pressure to a relative density of 1.04–1.06 (determined at 50°C), and centrifuged. The centrifuged solution was purified with HPD-100 macroporous resin to obtain a 50% alcohol eluent. The eluent was concentrated, concentrated to thick paste, and dried to obtain PLE. The extract was analyzed by high performance liquid chromatography to ensure quality standards. Chromatographic analysis conditions and representative HPLC chromatograms are shown in the Supplementary Figure 1.

### 2.3 Animal experimental design

**FIGURE 1:**
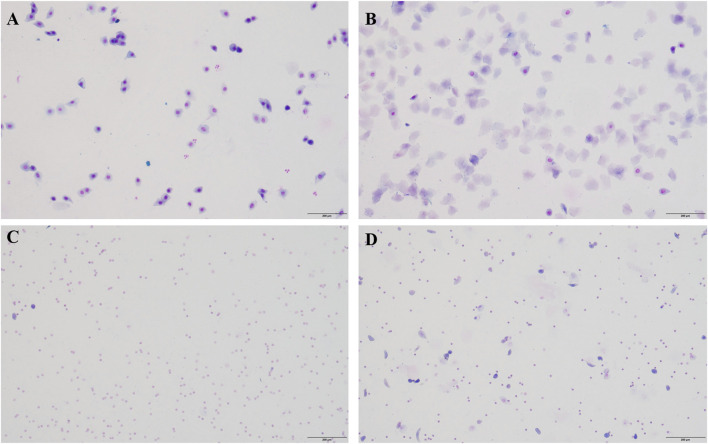
One complete erogenous cycle in normal sexually mature female rats. **(A)** Proestrus. During proestrus, a large number of nucleated epithelial cells can be seen, and sometimes a small number of keratinocytes can be seen. **(B)** estrus. During estrus, it was observed that the exfoliated cells were mainly anucleate keratinocytes, with few white blood cells. **(C)** late estrus. A large number of white blood cells appear in late estrus. **(D)** Diestrus. The exfoliated cells in the diestrus were mainly white blood cells, with a small amount of epithelial and keratinocyte cells. Bar = 200 μm.

Insufficiency of kidney yin leads to weakened physiological functions of the kidney, resulting in depletion of vital energy, significant damage to the primordial qi, and impairment of the Chong and Ren meridians, causing damage to the reproductive axis of the kidney - Tian Gui - Chong and Ren meridians - uterine cavity, and subsequently triggering various symptoms of climacteric syndrome (CMS). For instance, hot flashes. Methods that deplete kidney yin include excessive administration of thyroid hormones, adrenocorticotropic hormones, intragastric administration of warm and dry Chinese herbs, and overwork-induced yin deficiency. Previous research by our team has found that administration of thyroid tablet suspension (160 mg/kg) can induce hot flashes in menopausal rats. Therefore, in this study, we first established a CMS model using a natural aging model combined with the home invasion method, and then administered thyroid tablet suspension to induce hot flashes. After 1 week of adaptive feeding, the exfoliated vaginal cells of the rats were continuously monitored to determine a complete estrous cycle (5 days)(Li et al., 2019).The specific method is: use small cotton with physiological saline into the rat for vaginal exfoliated cells, besmear on glass slide, and observe under the microscope, and choose one which has an estrous cycle disordered for 5 days. A complete estrus cycle is early estrous, estrous, late estrous and estrous period (Figure 1).

Menopausal rats with irregular cycles were tested for ABT, EPM, OFT and surface temperature, tail temperature, and body weight. According to the experimental results, the menopausal rats were grouped according to the principle of uniformity. They were model group; Xiang Shao granule group (1.08 g/kg); Estrogen and Progesterone combination group (0.068 mg/kg + 0.42 mg/kg); PLE (40 mg/kg) group, PLE (80 mg/kg) group, PLE (160 mg/kg) group.

The main components of Xiangshao granules are: Radix paeoniae alba, Xiangfu, Chinaberry seed (fried), Bupleurum, Chuangxiong, Aurantii, Pinellia pinellia (ginger), cardamom, woody incense, licorice, etc,Prepare it as a suspension.The administration cycle was 28 days, and young rats were used as control group. On the 14th day of treatment, thyroid tablet suspension (160 mg/kg) was used to induce hot flashes symptoms in menopausal rats for 14 days. During the administration period, the survival status of rats should be observed daily, including compliance during gavage, hair loss, diet and drinking water, and urine and stool. The body weight of the rats was weighed and recorded weekly.

### 2.4 Behavioral tests

#### 2.4.1 Open field test (OFT)

The size of the open field experiment box is length × width × height = 50 cm × 50 cm × 40 cm. At the beginning of the experiment, the rats were placed in the center of the open field box. The activity of the rats in the box was recorded by the infrared camera system, and the XR-Super Maze animal behavior video tracking analysis system was used to analyze the motion trajectory of the synthetic rats. The recording time of each rat was set to 6 min (Cai et al., 2023; Gao et al., 2022). The observed indicators include the total distance of the rats, the center range of the rats, etc.

#### 2.4.2 Elevated plus maze (EPM)

The main observation box of the elevated cross maze is composed of an open arm of 50 cm × 10 cm × 40 cm, a closed arm, a central block of 10 cm × 10 cm × 40 cm and a base. At the beginning of the experiment, the rats were placed in the central block, and the video was recorded by the camera. Then the XR-Super Maze animal behavior video tracking and analysis system recorded the movement trajectory of the rats within 5 min and observed the behavior changes (Sun et al., 2023). Open Arm Entry (OE), Close Arm Entry (CE), Time in Open Arm (OT) and Time in Close Arm (CT) were observed. OE % and OT % were calculated according to the above observation indexes.

OE % = OE/(OE + CE) × 100%, OT % = OT/(OT + CT) × 100%.

#### 2.4.3 Aggressive behavior test (ABT)

Thirty minutes before the start of the experiment, the invasive mice were placed in the experimental mouse environment to adapt to the new environment. Put the experimental rat cage under the camera, remove the rat food and water bottles on the rat cage, so that the rat cage can be completely within the recording range of the camera. Put the invading rat in the cage, start the camera switch, start the video, the video time is 10 min, and record the fight in the cage within 10 min. After the start of the experiment, people are prohibited from walking and making noise, keeping quiet and reducing interference. After the experiment, the recorded video was analyzed using the rat aggressive behavior analysis software (Xiaoyuwang11, Ver 1.1). The analysis was blinded and evaluated by three trained personnel. The contents of the analysis include: attack (boxing, back top, pedal invasion of rats and other actions), climbing (the invasion of rats under the body, cannot be violent resistance), bite (mouth attack, bite the invasion of rats), vertical hair (rat hair erection) (Li et al., 2023; Liu et al., 2023; Wei et al., 2018).

#### 2.4.4 Surface temperature and tail temperature measurement

The left hand grasped and fixed the rat, exposed the rat’s face, and used a hand-held infrared temperature measuring gun to gently stick to the rat’s face to detect the facial skin temperature. The rat was fixed with the left hand. When the tail was stationary, the skin temperature at the upper 1/3 of the tail was measured three times in parallel, and the mean value was taken.

### 2.5 Enzyme-linked immunosorbent assay (ELISA)

The levels of E2 (Elabscience, E-OSEL-R0001), LH (Elabscience, E-EL-R0026c), FSH (Elabscience, E-EL-R0391c), 5-HT (CUSABIO, CSB-E08364r), cGMP (Nanjing Jiancheng, H163) and cAMP (Nanjing Jiancheng, H164) in serum were measured by ELISA kits. The serum was removed from the ultra-low temperature refrigerator 1 day before the formal experiment placed in a −20°C refrigerator for 6 h, and then transferred to a 4°C refrigerator to equilibrate overnight. The serum was centrifuged again before the start of the experiment for the official start of the experiment. The assay was performed with a full-wavelength enzyme marker. (Shanghai Meigu Molecular Instrument, SpectraMax iD5).

### 2.6 HE staining and uterine index

The uteruses fixed in 4% paraformaldehyde solution were dehydrated and embedded in paraffin to prepare 4 μm paraffin sections. After dewaxing, rehydration and other steps, HE staining was performed (Xiong et al., 2022). The images were photographed with an Olympus BX5-3 microscope.

$$\text{Uterine index}=\left(\text{uterine wet weight}/\text{body mass}\right)\;*\;100\%$$

### 2.7 Sample collection & sequencing of some samples

At the end of the treatment cycle, the rats were anesthetized with isoflurane and placed in a comatose state. The rat’s abdominal cavity was opened, and after blood was taken from the abdominal aorta the rat was decapitated and the hypothalamus was quickly separated on ice and weighed. Three hypothalamuses from each of the three groups, control group, model group and PLE (160 mg/kg) group,were sent to Shenzhen Huada Gene Technology Service Co. The original contributions presented in the study are publicly available. This data can be found here attachment.

The raw data obtained from sequencing was filtered using SOAP nuke (v1.5.6) to obtain clean data (Li et al., 2008). Clean data was compared to the reference genome using HISAT2 (v2.1.0) software (Kim et al., 2015). The clean data was compared to the reference gene set using Bowtie2 (v2.3.4.3) (Langmead and Salzberg, 2012). The reference gene set was provided by the Dr. Tom multi-omics data mining system. Gene expression quantification was performed using RSEM (v1.3.1) software (Li and Dewey, 2011). The analysis protocol was imported into the Dr. Tom multi-omics data mining system and differential gene detection between the control and model groups and between PLE (160 mg/kg) group and the model group was performed using DESeq2 (v1.4.5) with a condition of Q value ≤ 0.05 (Love et al., 2014).

### 2.8 Functional enrichment analysis of DEGs

The common differential genes between the control and model groups and between PLE (160 mg/kg) group and the model group were analyzed and visualized with the help of ggplot2 package and Venn Diagram package, and the obtained common differential genes were analyzed by heatmap package for hierarchical clustering, and for further in-depth exploration of the gene functions associated with phenotypic changes, we passed the obtained differential genes through Dr. Tom multi-omics data mining system for GO/KEGG pathway enrichment analysis (Wang W. et al., 2022).

### 2.9 Quantitative real-time PCR (qRT-PCR)

Total RNA was extracted from hypothalamic samples of all groups using the Fast Pure^®^ Cell/Tissue Total RNA Extraction Kit V2 (Vazyme, RC112), HiScript^®^ III RT Super Mix for qPCR (Vazyme, R323) was used for genomic DNA removal and reverse transcription reactions, and real-time quantitative PCR was performed using ChamQ SYBR qPCR Master Mix (Vazyme, Q311) on a high-resolution real-time fluorescent quantitative PCR instrument (QuantStudio3, Thermo Fisher, USA). Each sample was run in triplicates. Relative gene expressions were calculated using the 2^−ΔΔCT^ method. The primers (Table 1) were designed and synthesized by Beijing Prime Tech Biotechnology Co.

**TABLE 1:** Primers used in qRT-PCR.

Primer	Forward (5′–3′)	Reverse (5′–3′)
Asic4	TCCTGGTTGCCCTGAGTT	GCCTCCTTCTCCTTGGGT
Cplx1	CCTCTGGACACCGACCTT	TACGTGGCCCTCTGTCTG
Tac3	GGA​ACA​GCC​AAC​CAG​ACA​C	GGGGAGCCAACAGGAGGA
Tacr3	CTG​TTT​CCC​ACT​GCT​CAT​C	CCA​CCT​TTC​GTT​TAG​CCT​T
Kiss1	AGC​TGC​TGC​TTC​TCC​TCT​GT	AGG​CTT​GCT​CTC​TGC​ATA​CC
GnRH1	CCG​CTG​TTG​TTC​TGT​TGA​CTG​TG	GGG​GTT​CTG​CCA​TTT​GAT​CCT​C
β-actin	TGA​CCC​AGG​ACT​CTC​TCT​TCT​ATG​A	GAG​CGC​GTA​ACC​CTC​ATA​GAT

### 2.10 Western blot analysis

Total protein was extracted from hypothalamus using RIPA lysate (Bioss, C5029) and PMSF, and the concentration was determined using BCA protein concentration assay kit (Beyotime, P0010S), 30 μg samples of 10%–12.5% SDS-PAGE gel maintained at a constant voltage of 100 V for electrophoresis, and then the protein was transferred to PVDF membrane (0.22/0.45 μm, Millipore), 5% skim milk powder (1 × TBST preparation) closed in shaker for 3 h. 1 × TBST was washed 3 times in incubation box in shaker for 5 min each time. Primary antibody: GNRH1 (1:1000, A5625, Abclonal), CPLX1 (1:1000, 10246-2-AP, PTG), TACR3 (1:1000, bs-24783R, Bioss), TAC3 (1:1000, A6312, Abclonal), GAPDH (1:5000, 10494-1-AP, PTG), dilute primary antibody at the above ratio. Pour into the incubation kit and leave overnight at 4°C. The membrane was washed the next day and washed 5 times with TBST for 5 min. The diluted secondary antibody was added and incubated on a shaker for 1 h. The sample was developed with ECL Ultrasensitive Luminol (P90719, Millipore) and the bands were analyzed with ImageJ software to calculate the grayscale values of the bands.

### 2.11 Statistical methods

The experimental data were presented as the mean ± standard deviation (SD). The graphics in the article were performed with GraphPad prism 9.4 software. Comparisons of means among groups was performed by one-way ANOVA. A value of *P* < 0.05 was considered to be statistically significant.

## 3 Results

### 3.1 Effects of PLE on the changes of facial and tail temperature in rats with hot flashes

The rats in the control group were generally in good condition, with a normal diet, increasing body weight, neat and shiny hair, red and shiny tail and claw color, good spirit and sensitive response. Compared with the control group, the model rats, after 2 weeks of intragastric administration of thyroid tablets, showed obvious red tongue, increased tail temperature, dry feces, dry hair, irritability, difficulty in grasping, increased drinking water and diet. The mental state of rats treated with drug intervention has improved to a certain extent.

**FIGURE 2:**
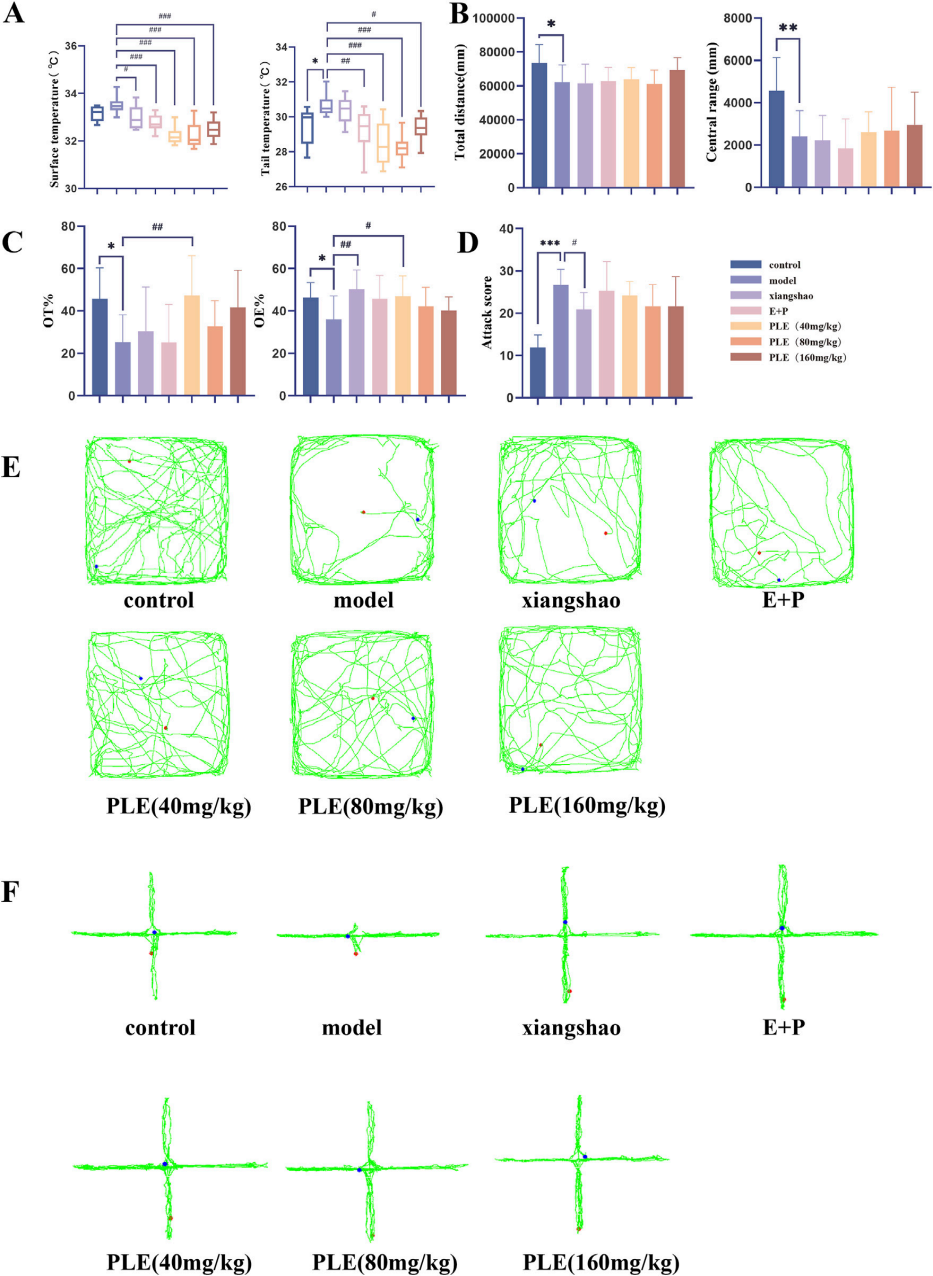
Effect of PLE on phenotypic changes in rats with hot flashes. **(A)** Comparison of surface temperature (left) and tail temperature (right) in each group. **(B)** Comparison of total distance (left) and central distance (right) in OFT. **(C)** Comparison of OT % (left) and OE % (right) in EPM. **(D)** Comparison of aggressive behaviour scores of rats in each group in ABT. **(E)** Representative trajectory map of OFT in each group. **(F)** Representative trajectory map of EPM in each group. Compared with the control group, *P < 0.05, **P < 0.01, ***P < 0.001; compared with the model group, ^#^ *P* < 0.05, ^##^ *P* < 0.01.

Compared with the control group (Figure 2A), the tail temperature of rats in the model group showed a significant increase after administration (*P* < 0.05), and the face temperature showed a tendency to increase after administration, but the difference was not statistically significant (*P* > 0.05). Compared with the model group (Figure 2A), the surface temperature of the rats in the Xiang Shao granule group, the Estrogen and Progesterone combination group, PLE (40 mg/kg) group, PLE (80 mg/kg) group, PLE (160 mg/kg) group were significantly reduced (*P <* 0.05, *P* < 0.001, *P* < 0.001, *P* < 0.001). Compared with the model group, the tail temperature of the rats in the estrogen and progesterone combined group, PLE (40 mg/kg) group, PLE (80 mg/kg) group, PLE (160 mg/kg) group were significantly decreased (*P <* 0.001, *P* < 0.001, *P* < 0.001, *P* < 0.001).

### 3.2 Effects of PLE on behavioral changes in rats with hot flashes

OFT, EPM and ABT were used to evaluate the effect of PLE on mood improvement in menopausal syndrome rats. In the OFT (Figures 2B,E), the total distance travelled in the open field and the distance travelled in the central zone were significantly lower in the model group rats compared with the control group (*P* < 0.05, *P* < 0.01); in the EPM (Figures 2C,F), the OE% and OT% values were significantly lower in the model group rats compared with the control group (*P* < 0.05, *P* < 0.05); in the ABT (Figure 2D), the aggressive behavior scores of rats in the model group were significantly higher compared with the control group (*P* < 0.01). It shows that the level of anxiety and irritability in the model group is higher, which is consistent with the clinical manifestations of anxiety and irritability in patients with hot flashes.

After drug treatment, there was no significant difference in the total distance travelled in the open field and the distance travelled in the central zone in each treatment group compared with the model group (*P* > 0.05); compared with the model group, the OE% of the EPM of rats in the Xiang Shao granule group and the PLE (40 mg/kg) group was significantly higher (*P* < 0.01, *P* < 0.05), and the OT% of the PLE (40 mg/kg) group was also significantly higher at the same time (*P* < 0.01). Compared with the model group, the aggressive behavior scores of rats in the aromatic Xiang Shao granule group were significantly lower (*P* < 0.05), and all other treatment groups showed a decreasing trend.

### 3.3 The effect of PLE on the content of E2, FSH, LH, cAMP, cGMP and 5-HT in serum of rats with hot flashes

**FIGURE 3:**
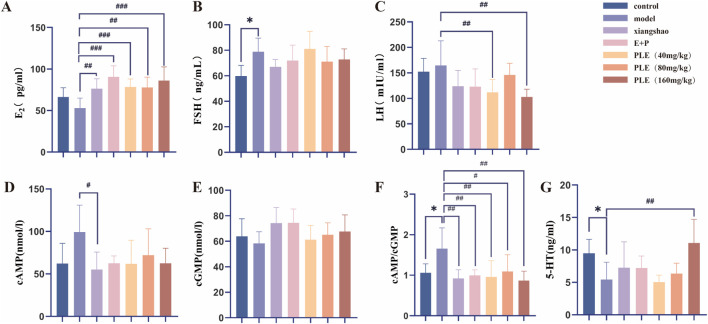
The effects of PLE on the content of E2, FSH, LH, cAMP, cGMP and 5-HT in serum of rats with hot flashes. **(A–G)** The contents of E2, FSH, LH, cAMP, cGMP, cAMP/cGMP and 5-HT in serum of rats in each group were compared. Compared with the control group, *P < 0.05; compared with the model group, ^#^ *P* < 0.05, ^##^ *P* < 0.01, ^###^ *P* < 0.001.

As shown in Figure 3A, compared with the control group, the serum E2 of the model group tended to decrease, but the difference was not statistically significant (*P* > 0.05). Compared with the model group, the serum E2 levels in the Xiang Shao granule group, the Estrogen and Progesterone combination group, PLE (40 mg/kg) group, PLE (80 mg/kg) group, PLE (160 mg/kg) group were significantly increased (*P* < 0.01, *P* < 0.001, *P* < 0.001, *P* < 0.01, *P* < 0.001). Compared with the control group (Figure 3B), the serum FSH of the model group was significantly increased (*P* < 0.05). Compared with the model group, although there was no significant decrease in each administration group, there was a decreasing trend. Compared with the control group (Figure 3C), the serum LH in the model group tended to increase, but the difference was not statistically significant (*P* > 0.05). Compared with the model group, the serum LH in the PLE (40 mg/kg) group and PLE (160 mg/kg) group was significantly lower (*P* < 0.01, *P* < 0.01).

Compared with the control group (Figure 3D), the serum cAMP in the model group showed an increasing trend, but the difference was not statistically significant (*P* > 0.05). Compared with the model group, the serum cAMP in the Xiang Shao granule group was significantly decreased (*P* < 0.05). Compared with the control group (Figure 3F), the serum cAMP/cGMP ratio of the model group was significantly increased (*P* < 0.05). Compared with the model group, the serum cAMP/cGMP ratio of Xiang Shao granule group, Estrogen and Progesterone combination group, PLE (40 mg/kg) group, PLE (80 mg/kg) group and PLE (160 mg/kg) group were significantly decreased (*P* < 0.01, *P* < 0.01, *P* < 0.01, *P* < 0.05, *P* < 0.01). Compared with the control group (Figure 3G), the serum 5-HT content of the model group was significantly decreased (*P* < 0.05). Compared with the model group, the content of serum 5-HT in the PLE (160 mg/kg) was significantly increased (*P* < 0.01).

### 3.4 Effects of PLE on the pathological changes of uterus tissue in rats with hot flashes

**FIGURE 4:**
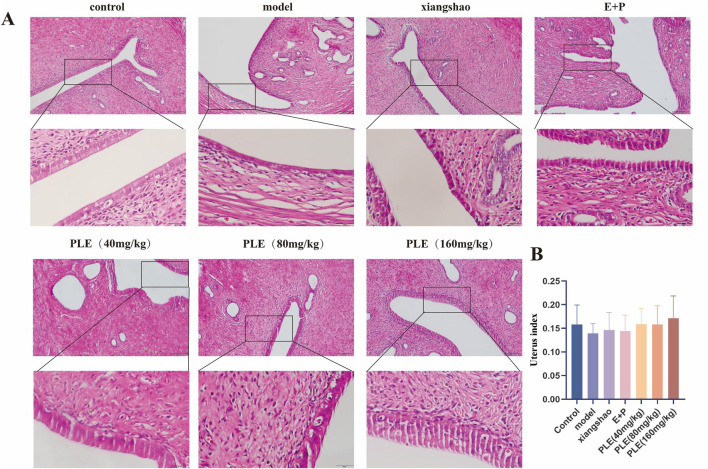
Effects of PLE on the pathological changes of uterus tissue in rats with hot flashes. **(A)** Representative images of HE staining of rat uterus. Bar = 200 μm. (upper), Bar = 200 μm. (lower). **(B)** Comparison of uterus index in each group of rats.

The uterine structure of the rats in the control group was normal, which was composed of endometrium, muscular layer and serous layer (Figure 4A). The endometrial epithelium is a single columnar, with a small number of ciliated cells and a large number of non-ciliated secretory cells. The lamina propria is rich in stromal cells, glands and blood vessels. The muscular layer is thicker, composed of abundant smooth muscle bundles and blood vessels, and there are connective tissue intervals between the bundles. The uterus of rats in the model group showed atrophic thinning, endometrial atrophy and thinning, tissue structure disorder, loose arrangement of muscle fibers, thinning of the muscle layer, atrophy and decrease of uterine glands and capillaries. Compared with the model group (Figures 4A,B), the uterine volume of the treated rats increased, the endometrium thickened, the tissue structure was arranged more regularly, the glands and capillaries increased and increased, the muscle fibers were arranged more consistently, and the muscle layer was thickened.

### 3.5 Transcriptomic analysis of the potential targets of PLE for the treatment of menopausal hot flashes

With the help of the DNBSEQ platform of BGI, nine samples were detected, with an average output of 6.79 G data per sample. Through the comparison of reference genes and reference gene sets, the average comparison rates of samples were 92.91% and 62.27%, respectively. Finally, 19,351 genes were obtained by statistical analysis. At the same time, the sequencing results showed that the sequencing quality and library establishment of this study were good without any pollution, which provided a basis for further analysis.

**FIGURE 5:**
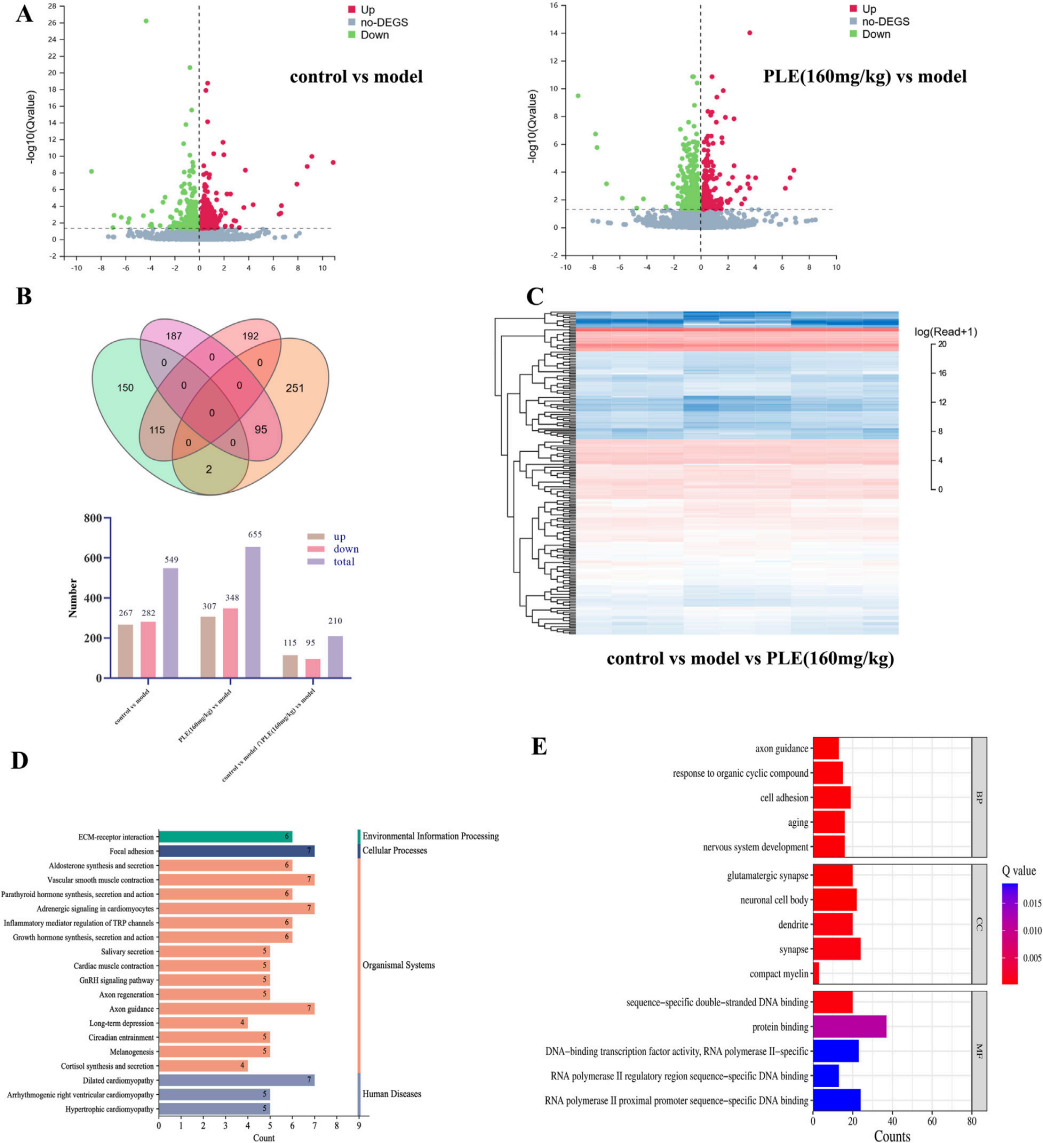
Transcriptomic analysis of the potential targets of PLE for the treatment of menopausal hot flashes **(A)** The volcano plot of control vs. model (left), the volcano plot of PLE (160 mg/kg) vs. model (right), red represents an increase, green represents a decrease; **(B)** control vs. model vs. PLE (160 mg/kg). **(C)** Control vs. model vs. PLE (160 mg/kg) common differential mRNA heat map, red represents high expression, blue represents low expression; **(D)** KEGG pathway enrichment analysis of common differential mRNA; **(E)** GO enrichment analysis of common differential mRNA, BP represents Biological Process, CC represents Cellular Component and MF represents Molecular Function.

It can be seen from the volcano, Veen and heatmap of differential mRNA that compared with the model group, 267 genes were significantly upregulated and 282 genes were significantly downregulated in the control group. There were 307 upregulated genes and 348 downregulated genes in the PLE (160 mg/kg) group. There were 115 upregulated genes and 95 downregulated genes in the control group and the PLE (160 mg/kg) group (Figures 5A–C). The results showed that the gene expression in the hypothalamus of experimental animals was significantly changed by modeling and treatment.

The KEGG database systematically analyzes the annotated pathways of genes by combining gene, genome information and higher-level functional information. The phyper function in R software is used for enrichment analysis to calculate *P* value, and then FDR correction is performed on *P* value to obtain Q value. Usually, functions with Q value ≤ 0.05 are regarded as significant enrichment. The KEGG enrichment pathway of common differential genes in the control group and the PLE (160 mg/kg) group mainly involved 213 pathways. It mainly includes GnRH signaling pathway, Axon regeneration, Axon guidance, Long-term depression, Glutamatergic synapse, GABAergic synapse, Calcium signaling pathway, Dopaminergic synapse, Neuroactive ligand-receptor interaction, Estrogen signaling pathway and other pathways. The GO database includes three categories: Biological Process, BP, Cellular Component, CC and Molecular Function, MF, which describe the possible molecular functions of gene products, the cellular environment in which they are located, and the biological processes involved. GO enrichment found that these differentially expressed proteins were mainly concentrated in axon guidance, aging, nervous system development, glutamatergic synapse, neuronal cell body, synapse, protein binding, etc. We found that most of the differentially expressed genes play an important role in regulating neurological function. Among them, GnRH signaling pathway and Neuroactive ligand-receptor interaction are the signaling pathways that we have focused on in the early stage.

**FIGURE 6:**
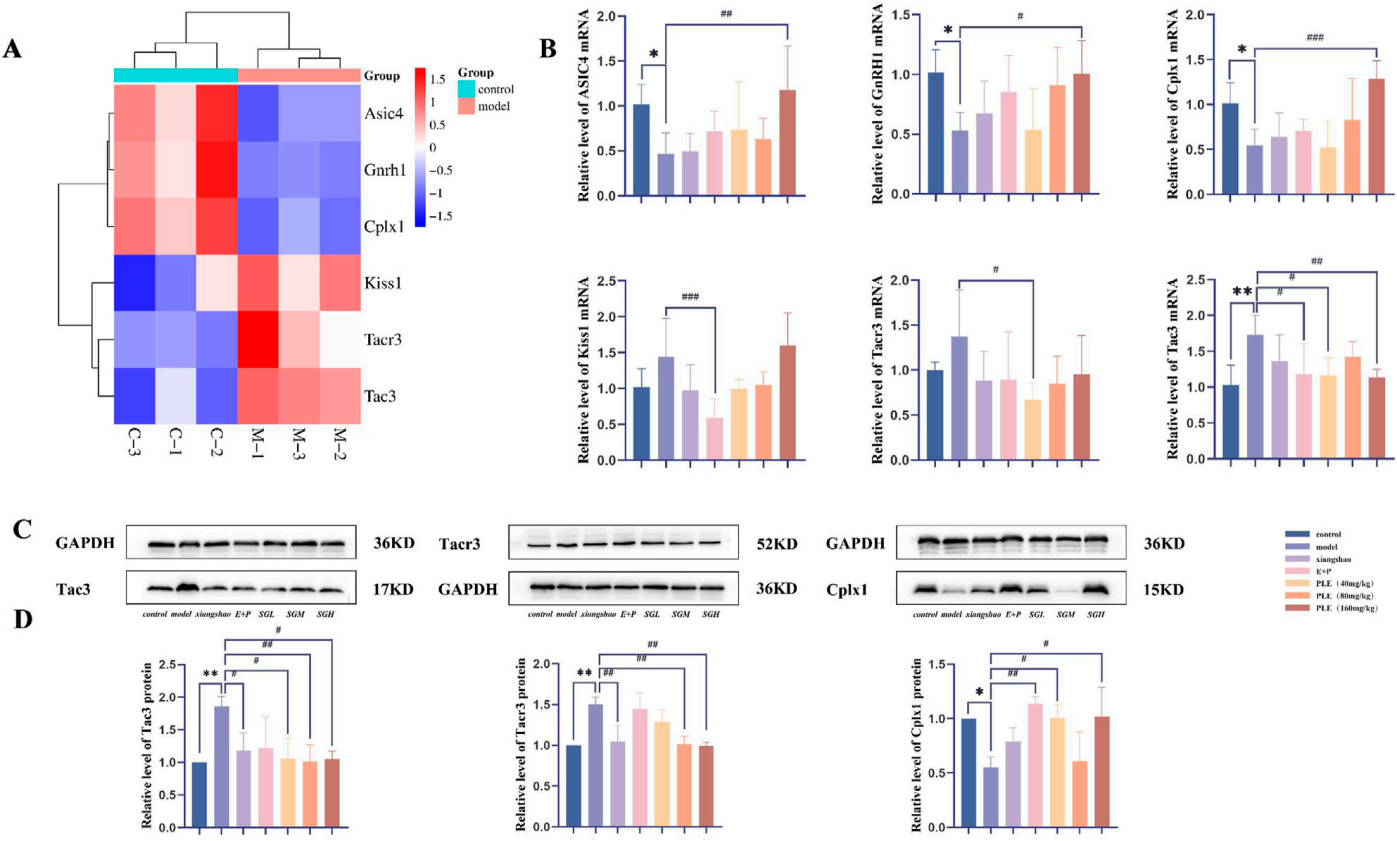
qRT-PCR and Western blot were used to analyze the mRNA and protein expression of candidate genes in the hypothalamus of rats in each group. **(A)** Count heatmap of candidate genes in rat hypothalamus in control and model, red represents high expression, blue represents low expression; **(B)** The relative expression levels of ASIC4, Cplx1, GnRH1, Kiss1, Tac3 and Tacr3 mRNA in hypothalamus were detected by qRT-PCR. **(C)** Western blot was used to detect the representative pictures of Tacr3, Tac3 and Cplx1 protein expression in hypothalamus; **(D)** Western blot was used to detect the relative expression levels of Tacr3, Tac3 and Cplx1 proteins in the hypothalamus. Compared with the control group, *P < 0.05, **P < 0.01; compared with the model group, ^#^ *P* < 0.05, ^##^ *P* < 0.01.

We further focused on genes related to neural pathways. The heat maps of these genes are shown in Figure 6A, including ASIC4 belonging to the ASIC gene family of neuronal proton-gated cation channels, neurotransmitter release regulatory protein cplx1 in the presynaptic structure, GnRH1 in the GnRH signaling pathway, and Tac3, Kisspeptin and Tacr3 in the KNDy neuron cluster. The transcriptional expression profiles of these genes in the model group and the control group changed significantly, and were significantly reversed after treatment with PLE.

### 3.6 Verification of candidate mRNA by qRT-PCR

Compared with the control group, the expression levels of ASIC4, Cplx1 and GnRH1 mRNA in the hypothalamus of the model group were significantly decreased (*P* < 0.05), the expression level of Tac3 mRNA was significantly increased (*P* < 0.01), and the expression levels of Kiss1 and Tacr3 mRNA were increased, but the difference was not statistically significant (*P* > 0.05). After drug intervention, it can be clearly seen that each index has a callback trend. The expression levels of ASIC4, Cplx1 and GnRH1 mRNA in the hypothalamus of rats in the PLE (160 mg/kg) group were significantly higher than those in the model group (*P* < 0.01, *P* < 0.001, *P* < 0.05). The expression levels of Kiss1 and Tac3 mRNA in the hypothalamus of rats in the Estrogen and Progesterone combination group were significantly lower than those in the model group (*P* < 0.001, *P* < 0.05). The expression levels of Tac3 and Tacr3 mRNA in the hypothalamus of the PLE (40 mg/kg) group were significantly lower than those in the model group (*P* < 0.05, *P* < 0.05). The expression level of Tac3 mRNA in the hypothalamus of the PLE (160 mg/kg) group was significantly lower than that in the model group (*P* < 0.01).

### 3.7 Effects of PLE on protein expression of cplx1, Tac3, and Tacr3 in the hypothalamus

As shown in Figures 6C,D, compared with the control group, the expression level of Cplx1 protein in the hypothalamus of the model group was significantly decreased (*P* < 0.05), and the expression levels of Tac3 and Tacr3 proteins were significantly increased (*P* < 0.01, *P* < 0.01). After drug intervention, it can be clearly seen that all indicators have a callback trend. Compared with the model group, the expression levels of Tac3 and Tacr3 proteins in the hypothalamus of the Xiang Shao granule group were significantly reduced (*P* < 0.01, *P* < 0.05). The expression of Cplx1 protein in hypothalamus was significantly increased in the Estrogen and Progesterone combination group (*P* < 0.01). The expression levels of Tac3 protein in hypothalamus of rats in PLE (40 mg/kg) group were significantly decreased (*P* < 0.05), and the expression level of Cplx1 protein was significantly increased (*P* < 0.05). The expression levels of Tac3 and Tacr3 protein in hypothalamus of rats in the PLE (80 mg/kg) group were significantly decreased (*P* < 0.01, *P* < 0.01). The expression levels of Tac3 and Tacr3 protein in hypothalamus of rats in the PLE (160 mg/kg) group were significantly decreased (*P* < 0.05, *P* < 0.01), and the expression level of Cplx1 protein was significantly increased (*P* < 0.05).

## 4 Discussion

The pathological mechanism of hot flashes is very complex and its development includes estrogen withdrawal, abnormal neurotransmitter metabolism, sympathetic nervous system dysregulation, and abnormal neuronal activity in the brain (Fleischer et al., 2021; Sun et al., 2022). The mainstay of treatment is HRT (Talaulikar, 2022). Although effective in relieving menopause-related symptoms, HRT has many contraindications and adverse effects, making it a deterrent for some patients (Zhang et al., 2020). Traditional Chinese medicine has rich experience as well as significant advantages in the clinical treatment of hot flashes, with stable and reliable efficacy and few toxic side effects (Song et al., 2022; Zhang et al., 2020).

*Radix Paeoniae Alba* (Bai Shao), a commonly used traditional Chinese medicine, has the effects of nourishing blood, regulating menstruation, astringing Yin and stopping sweating, softening the liver and relieving pain, and calming liver Yang, and is used for blood deficiency and yellowing, irregular menstruation, spontaneous sweating, night sweating, dysmenorrhea, abdominal pain, painful contracture of limbs, headache and dizziness (Commission, 2020). PLE is extracted from *Radix Paeoniae Alba*, refined, concentrated and dried to produce a natural extract. It has good pharmacological activities such as anti-inflammatory, anti-aging, antispasmodic and analgesic, cardiovascular protection, nervous system protection and immunomodulation (Kim et al., 2021; Lee et al., 2022; Wang et al., 2020; Wang L. et al., 2022). Here, the effects of PLE anti-menopausal hot flashes on thyroid tablet-induced changes in facial and tail temperature as well as behavior in menopausal rats and the mechanism of their neuroprotective effects were investigated.

In this study, the menopausal hot flashes rat model was prepared by gavage of thyroid tablets in naturally aging rats. The model was stable and could better simulate the clinical manifestations of yin deficiency, hot flashes, sweating and irritability in menopausal patients. The results showed that the model rats showed the basic symptoms of anxiety and irritability and elevated tail temperature from the physiological indications, and the administration of PLE could effectively improve the symptoms of irritability, anxiety and elevated tail temperature in the model rats.

In addition, from the serum microscopic indexes, the serum levels of FSH and cAMP/cGMP ratio in the model rats were significantly higher than those in the control group, while the 5-HT content was significantly lower than that in the control group. After the administration of PLE, the serum levels of sex hormones, neurotransmitters and cAMP/cGMP ratio in the model rats were effectively regulated. From the pathological microscopic indexes, the uterus of the model rats was obviously shrunken, the endometrium was thinner and the tissue fibers were loosely arranged, while the uterus of the treated rats increased in volume, the endometrium was thickened, the tissue structure was more regularly arranged, the glands and capillaries were larger and increased, the muscle fibers were more uniformly arranged, and the muscle layer was thickened. The above indicates that the model was successfully modeled and PLE has a good pharmacological basis for improving the symptoms of hot flashes in the model rats.

Recent evidence suggests that the occurrence of hot flashes is related to the hypothalamic neural circuits that control GnRH secretion (Barlow, 2023; Padilla et al., 2018). By analyzing the transcriptome sequencing results through Dr. Tom’s multi-omics data mining system, we compared the gene expression characteristics of hypothalamic tissue in the model, control, and PLE (160 mg/kg) groups of rats, and obtained genes associated with the onset of menopausal hot flashes and those altered in the hypothalamus of model rats after the administration of intervention. We further focused on the genes related to neural pathways, among which ASIC4, Cplx1, GnRH1, Kiss1, Tac3, Tacr3 were of great interest.

Asic4 belongs to the ASIC gene family of neuronal proton-gated cation channels, which are widely expressed in the mammalian central nervous system (Hoshikawa et al., 2017). The acid-sensitive ion channel family was found to be involved in regulating vasodilatory responses under normal or low pH conditions, as well as maintaining resting tone during diastole induced by acidosis (Akanji et al., 2019). In addition, one study identified ASIC4 as a key downstream target gene regulated by FST, Follistatin mediates learning and synaptic plasticity via regulation of Asic4 expression in the hippocampus (Chen et al., 2021). Cplx1 is an important neurotransmitter release regulatory protein in presynaptic structures that forms the SNARE complex in the CNS and is involved in the anchoring, prestimulation and fusion of axon terminal vesicles. Neurodegenerative and psychiatric disorders are correlated with abnormal expression of Cplx1(Jesko et al., 2021; Xu et al., 2020). Specific, widely-scattered neurons in the hypothalamus secrete the decapeptide GnRH1 in an episodic pattern of pulses. It is secreted in a pulsatile manner into the pituitary portal system to the anterior pituitary, where it binds to receptors on pituitary gonadotropin cells (Ferris and Shupnik, 2006). This promotes the synthesis and secretion of LH and FSH, which in turn act on the gonads through the peripheral blood circulation. The pulsatile release of GnRH plays an important role in the regulation of animal reproduction (Casati et al., 2023; Ortmann et al., 2002). Furthermore, it has also been found that neuronal populations in the hypothalamus expressing both Kisspeptin/Tac3/Dynorphin directly regulate GnRH secretion and thus pituitary gonadotropin secretion, and that these neurons also project to the vasodilatory center, which is over-activated in estrogen deficiency leading to hot flashes in menopausal women (Nagae et al., 2021; Rance et al., 2013; Szeliga et al., 2018).

From the experimental results, Asic4, Cplx1 and GnRH1 mRNA were significantly downregulated and Tac3 mRNA was significantly upregulated in the hypothalamus of the model rats compared with the control group, which was consistent with the transcriptomic results. Gene expression levels were adjusted and hot flashes improved after administration of therapeutic drugs, presumably as a possible target for the action of PLE in the treatment of menopausal hot flashes. Further studies on protein revealed that hypothalamic Tac3 and Tacr3 protein levels were significantly upregulated and Cplx1 protein levels were significantly downregulated in the model rats compared to the control group. However, the treatment with PLE reversed the upregulation of Tac3 and Tacr3 protein levels and the downregulation of Cplx1 protein levels, suggesting that PLE helps to inhibit the over-activation of Tac3 neuronal cell populations, thereby regulating the entire neural circuit and hormonal circulation *in vivo*.

## 5 Conclusion

In this study, a menopausal hot flashes rat model was successfully established. PLE, as a traditional Chinese medicine extract, may improve the symptoms of anxiety and irritability as well as hot flashes in model rats by inhibiting the activation of Tac3 neurons, decreasing Tacr3 expression and increasing Cplx1 expression, thus coordinating the response of hypothalamic neural circuits and increasing E2 and 5-HT levels *in vivo*. However, the therapeutic effects of PLE on other aspects, especially the deeper mechanisms of treatment, need to be further investigated.

### 5.1 Strengths and limitations

Based on previous research, this study proposed a new rat model of menopausal hot flashes. It was found that hot flashes induced by thyroid tablets in menopausal rats exhibited similar clinical symptoms and histological changes to those in menopausal women, laying a foundation for the study of menopausal hot flashes. However, whether the menopausal hot flash rat model can fully replicate the real clinical symptoms of menopausal hot flash women and whether thyroid tablets will cause changes in thyroid function still need further comprehensive research. In addition, the hypothalamus, which is the center of the endocrine and nervous systems, often shows significant changes in patients with hot flashes. Therefore, studying the changes in the hypothalamus is crucial for animal models. However, due to the insufficiency of detection methods, the potential pathogenesis of menopausal hot flashes remains unclear, and further research is needed in the future.

## Data Availability

The datasets presented in this study can be found in online repositories. The names of the repository/repositories and accession number(s) can be found in the article/Supplementary Material.

## Abbreviations

PLE, Paeonia Lactiflora Extract; E + P, Estradiol valerate + Progesterone; OFT, Open Field Test; EPM, Elevated Plus-maze test; ELISA, Enzyme-linked immunosorbent assay; E2, Estradiol; FSH, Follicle Stimulating Hormone; LH, Luteinizing Hormone; cAMP, Cyclic adenosine monophosphate; cGMP, Cyclic guanosine monophosphate; HE, Hematoxylin-eosin staining; qRT-PCR, Quantitative real-time PCR.
