# Supplementary figures and images for: Therapeutic effects and mechanism analysis of Paeonia lactiflora extract (PLE) in menopausal rats with hot flashes
Source: Front Pharmacol. 2025 Jul 22;16:1587885. doi: 10.3389/fphar.2025.1587885 (PMC12321899; doi:10.3389/fphar.2025.1587885)

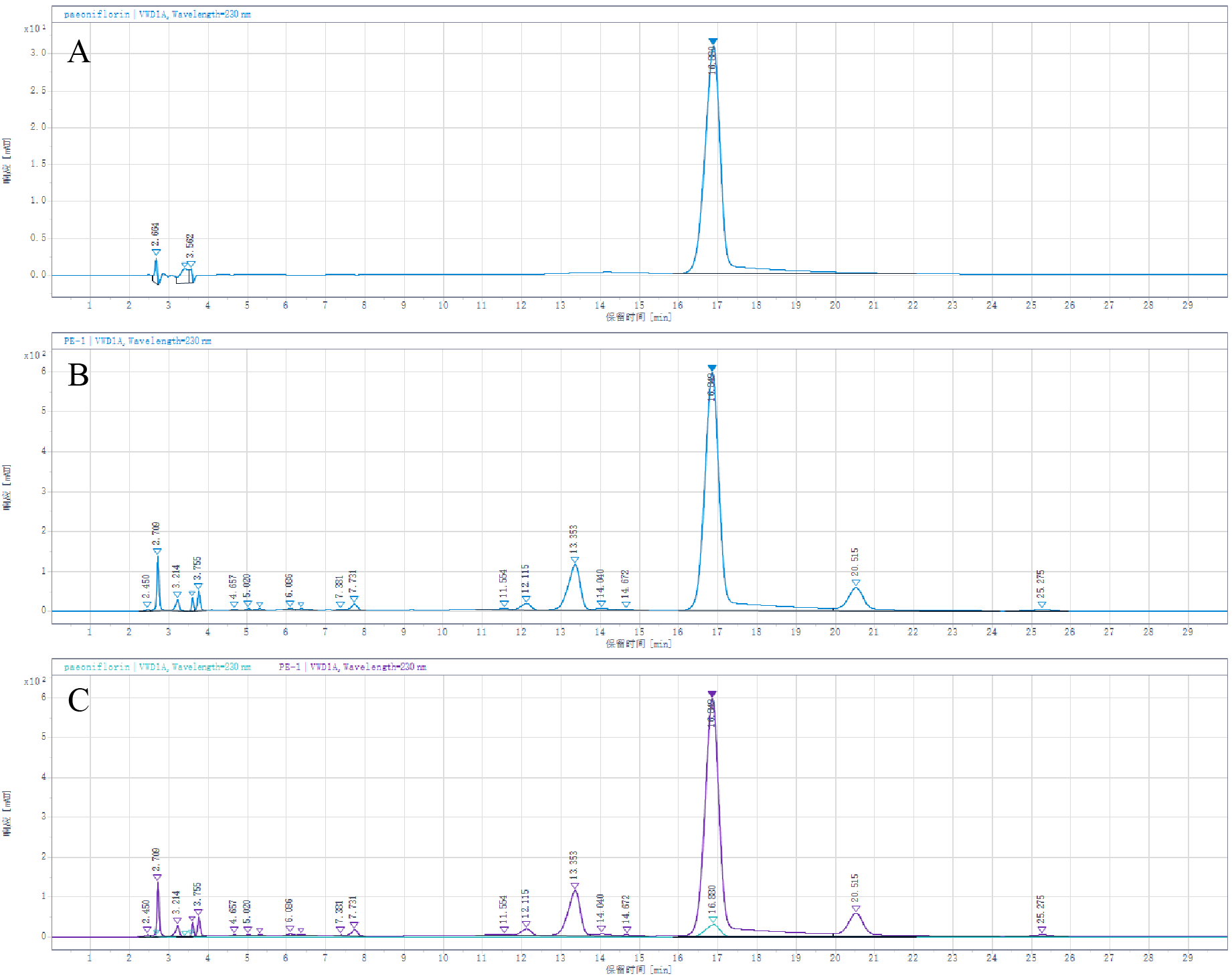

Supplement: Supplementary file 3 [file Image1.tif]
